# Correction: Variability in engagement and progress in efficacious integrated collaborative care for primary care patients with obesity and depression: Within-treatment analysis in the RAINBOW trial

**DOI:** 10.1371/journal.pone.0238276

**Published:** 2020-08-21

**Authors:** Nan Lv, Lan Xiao, Marzieh Majd, Philip W. Lavori, Joshua M. Smyth, Lisa G. Rosas, Elizabeth M. Venditti, Mark B. Snowden, Megan A. Lewis, Elizabeth Ward, Lenard I. Lesser, Leanne M. Williams, Kristen M. J. Azar, Jun Ma

The eleventh author's name is spelled incorrectly. The correct name is: Lenard I. Lesser.
