# Supplementary material for: Variability in engagement and progress in efficacious integrated collaborative care for primary care patients with obesity and depression: Within-treatment analysis in the RAINBOW trial
Source: PLoS One. 2020 Apr 21;15(4):e0231743. doi: 10.1371/journal.pone.0231743 (PMC7173791; doi:10.1371/journal.pone.0231743)
Supplement: S1 Appendix — a In-between session support as needed via EHR secure email, between weeks 1–52. b Co-located psychiatric and medical supervision during weekly intervention management team meeting, between weeks 1–52. c The 9 one-on-one I-CARE sessions will occur primarily in the clinic, but video conferences (as the second option) or phone sessions (as the last option and for visits 1–5, phone session is only an option upon PI and intervention manager approval) throughout the intensive phase will be an option for participants with considerable constraints. d I-CARE Mood is the PEARLS program; I-CARE Lifestyle is the GLB program. e Participants receive Fitbit, MyFitnessPal, and My Health Online instructions via mail or e-mail prior to first session. (DOCX) [file pone.0231743.s001.docx]

**S1 Appendix. Intervention Outline**^a,b^

| **Week** | **Visit**^c^ | **Minutes** | **Content** |
| --- | --- | --- | --- |
| 1 | 1 | 60 | - Introduction to I-CARE Mood and I-CARE Lifestyle^d^ sequenced integration (5 min) - Technology: Fitbit, MyFitnessPal - using, linking, friend requests to coach; My Health Online.^e^ Encouragement to wear Fitbit and check uploads (10 min) - Physical activity safety guidelines handout (GLB Session 4, pages 3,4) - Evaluation of bathroom scale ownership - I-CARE Mood Session 1 (45 min) |
| 2 | 2 | 60 | - I-CARE Mood Session 2 (60 min) - Provision of bathroom scale to participants requiring one |
| 3 | 3 | 60 | - I-CARE Mood Session 3 (60 min) |
| 4 | 4 | 60 | - I-CARE Mood Session 4 (50 min) - Technology: MyFitnessPal - explanation of logging physical activity minutes and weight; introduction to logging diet; importance of self-monitoring (10 min) |
| 6 | 5 | 60 | - I-CARE Mood Session 5 (45 min) - I-CARE Lifestyle introduction and self-study guidelines (5 min) - Technology: MyFitnessPal - review of logging physical activity and weight; explanation of logging diet; importance of self-monitoring (10 min) |
| 8 | 6 | 60 | - I-CARE Lifestyle Progress Check (5 min) - I-CARE Mood Session 6 (30 min) - Technology: MyFitnessPal , Fitbit, My Health Online check in (5 min) - Goals confirmation: weight, physical activity, steps (5 min) - Optional tools to reduce calorie intake: meal plans, packaged meals (5 min) - I-CARE Lifestyle Session (GLB Sessions #1,2) (10 min) |
| 12 | 7 | 60 | - I-CARE Lifestyle Progress Check (10 min) - I-CARE Mood Session 7 (35 min) - I-CARE Lifestyle Session (GLB Sessions #3,4,5,6) (15 min) |
| 16 | 8 | 60 | - I-CARE Lifestyle Progress Check (10 min) - I-CARE Mood Session 8 (35 min) - I-CARE Lifestyle Session (GLB Sessions #8,9,10)(15 min) |
| 20 | 9 | 60 | - I-CARE Lifestyle Progress Check (10 min) - I-CARE Mood Session 9 (35 min) - I-CARE Lifestyle Session (GLB Sessions #11,12)(10 min) - Overview of weeks 21-52 (5 min) |
| 21-52 | Phone | 15-30 | - I-CARE Lifestyle Progress Check (5-10 min) - I-CARE Mood Session(5-10 min) - I-CARE Lifestyle Session (5-10 min) - Discussion of maintenance plan when program goals met |

^a^ In-between session support as needed via EHR secure email, between weeks 1-52.

^b^ Co-located psychiatric and medical supervision during weekly intervention management team meeting, between weeks 1-52.

^c^ The 9 one-on-one I-CARE sessions will occur primarily in the clinic, but video conferences (as the second option) or phone sessions (as the last option and for visits 1-5, phone session is only an option upon PI and intervention manager approval) throughout the intensive phase will be an option for participants with considerable constraints.

^d^ I-CARE Mood is the PEARLS program; I-CARE Lifestyle is the GLB program.

^e^ Participants receive Fitbit, MyFitnessPal, and My Health Online instructions via mail or e-mail prior to first session.
