# Supplementary material for: Variability in engagement and progress in efficacious integrated collaborative care for primary care patients with obesity and depression: Within-treatment analysis in the RAINBOW trial
Source: PLoS One. 2020 Apr 21;15(4):e0231743. doi: 10.1371/journal.pone.0231743 (PMC7173791; doi:10.1371/journal.pone.0231743)
Supplement: S2 Appendix — (DOCX) [file pone.0231743.s002.docx]

**S2 Appendix. Individual participant trajectories within each cluster of percent weight change**

| 1. **Individual participant trajectories within cluster 1**   **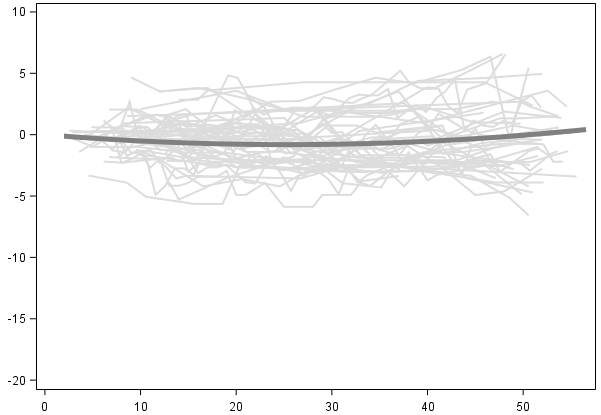** |
| --- |
| 1. **Individual participant trajectories within cluster 2**   **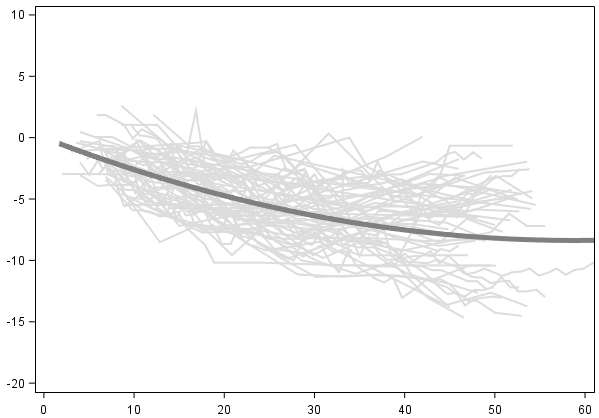** |
| 1. **Individual participant trajectories within cluster 3**   **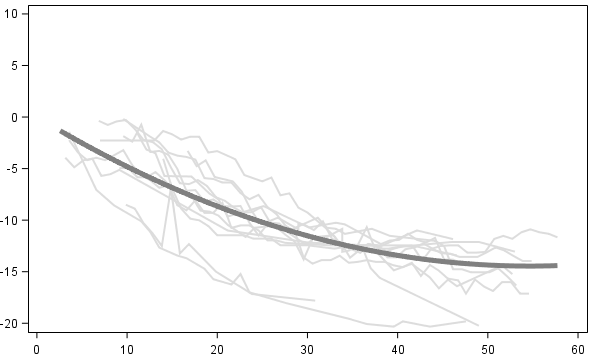** |
