# Supplementary material for: Variability in engagement and progress in efficacious integrated collaborative care for primary care patients with obesity and depression: Within-treatment analysis in the RAINBOW trial
Source: PLoS One. 2020 Apr 21;15(4):e0231743. doi: 10.1371/journal.pone.0231743 (PMC7173791; doi:10.1371/journal.pone.0231743)
Supplement: S3 Appendix — β1 = Linear coefficient; β2 = Quadratic coefficient. abcDifferent letters indicate significant difference. (DOCX) [file pone.0231743.s003.docx]

**S3 Appendix. Mean (**±**SD) beta coefficients of individual trajectories within each cluster of percent weight change**

|  | **Cluster 1-Minimal weight loss** (n = 50) | **Cluster 2-Moderate weight loss** (n = 61) | **Cluster 3-Most weight loss** (n = 12) | ***P* value** |
| --- | --- | --- | --- | --- |
| β1 | -0.03 ± 0.17^a^ | -0.30 ± 0.19^b^ | -0.56 ± 0.27^c^ | <0.001 |
| β2 | 0.0005 ± 0.005^a^ | 0.003 ± 0.005^b^ | 0.005 ± 0.006^b^ | 0.003 |

β1 = Linear coefficient; β2 = Quadratic coefficient.

^abc^Different letters indicate significant difference.
