# Supplementary material for: Variability in engagement and progress in efficacious integrated collaborative care for primary care patients with obesity and depression: Within-treatment analysis in the RAINBOW trial
Source: PLoS One. 2020 Apr 21;15(4):e0231743. doi: 10.1371/journal.pone.0231743 (PMC7173791; doi:10.1371/journal.pone.0231743)
Supplement: S5 Appendix — β0 = Intercept; β1 = Linear coefficient; β2 = Quadratic coefficient. abcDifferent letters indicate significant difference. (DOCX) [file pone.0231743.s005.docx]

**S5 Appendix. Mean (±SD) beta coefficients of individual trajectories within each cluster of PHQ-9 change**

|  | **Cluster 1-Moderate depression without treatment progress** (n = 40) | **Cluster 2-Moderate depression with treatment progress** (n = 20) | **Cluster 3-Milder depression with treatment progress** (n = 81) | ***P* value** |
| --- | --- | --- | --- | --- |
| β0 | 11.13 ± 2.95^a^ | 13.10 ± 3.05^b^ | 7.43 ± 2.44^c^ | <0.001 |
| β1 | -0.14 ± 0.35^a^ | -0.47 ± 0.34^b^ | -0.25 ± 0.25^c^ | <0.001 |
| β2 | 0.003 ± 0.01^a^ | 0.007 ± 0.008^a^ | 0.004 ± 0.006^a^ | 0.17 |

β0 = Intercept; β1 = Linear coefficient; β2 = Quadratic coefficient.

^abc^Different letters indicate significant difference.
