# Supplementary material for: Variability in engagement and progress in efficacious integrated collaborative care for primary care patients with obesity and depression: Within-treatment analysis in the RAINBOW trial
Source: PLoS One. 2020 Apr 21;15(4):e0231743. doi: 10.1371/journal.pone.0231743 (PMC7173791; doi:10.1371/journal.pone.0231743)
Supplement: S6 Appendix — Abbreviations: BMI, body mass index; PHQ-9, Patient Health Questionnaire-9; SCL20, Symptom Checklist-20. Values are mean (SD) unless otherwise noted. (DOCX) [file pone.0231743.s006.docx]

**S6 Appendix. Comparisons of baseline characteristics by category of treatment engagement and progress**

| **Characteristic** | **Poor engagement**  **(n=63; 31%)** | **Poor progress**  **(n=80; 39%)** | **Progress**  **(n=61; 30%)** | ***P* value** |
| --- | --- | --- | --- | --- |
| Age, year | 50.2 (12.9) | 49.4 (12.3) | 53.7 (11.1) | 0.10 |
| Female, No. (%) | 44 (70) | 63 (79) | 37 (61) |  |
| Race/Ethnicity, No. (%) |  |  |  | 0.21 |
| Non-Hispanic White | 42 (67) | 56 (70) | 49 (80) |  |
| Minority | 21 (33) | 24 (30) | 12 (20) |  |
| Education, No. (%) |  |  |  | 0.052 |
| High school to some college | 22 (35) | 23 (29) | 16 (27) |  |
| College graduate | 17 (27) | 40 (50) | 21 (34) |  |
| Post college | 24 (38) | 17 (21) | 24 (39) |  |
| Income, No. (%), n=176 |  |  |  | 0.07 |
| <$100,000 | 20 (36) | 31 (46) | 15 (28) |  |
| $100,000- <$150,000 | 9 (16) | 16 (24) | 9 (17) |  |
| ≥$150,000 | 26 (47) | 20 (30) | 30 (56) |  |
| Marital status, No. (%), n=203 |  |  |  | 0.11 |
| Married/living with a partner | 39 (62) | 42 (53) | 42 (70) |  |
| Single/separated/divorced/widowed | 24 (38) | 38 (48) | 18 (30) |  |
| Household size, No. (%), n=203 |  |  |  | 0.60 |
| < 2 | 13 (21) | 18 (23) | 9 (15) |  |
| = 2 | 21 (34) | 26 (33) | 27 (44) |  |
| 3+ | 28 (45) | 36 (45) | 25 (41) |  |
| BMI, kg/m^2^ | 36.3 (6.8) | 37.2 (6.4) | 36.5 (7.6) | 0.69 |
| PHQ-9 | 14.6 (3.2) | 14.1 (3.3) | 13.7 (3.1) | 0.26 |
| SCL-20 | 1.5 (0.6) ab | 1.6 (0.5) a | 1.3 (0.5) b | 0.03 |

Abbreviations: BMI, body mass index; PHQ-9, Patient Health Questionnaire-9; SCL20, Symptom Checklist-20.

Values are mean (SD) unless otherwise noted.

^a, b^ Different superscripts denote statistically significant differences between categories.
