# Supplementary material for: Variability in engagement and progress in efficacious integrated collaborative care for primary care patients with obesity and depression: Within-treatment analysis in the RAINBOW trial
Source: PLoS One. 2020 Apr 21;15(4):e0231743. doi: 10.1371/journal.pone.0231743 (PMC7173791; doi:10.1371/journal.pone.0231743)
Supplement: S7 Appendix — Abbreviations: NA, not applicable (SD is NA because only 1 participant attended session 9 in the poor engagement category). a, b, c Different superscripts denote statistically significant differences between categories. (DOCX) [file pone.0231743.s007.docx]

**S7 Appendix. Mean (±SD) number of days from session 1 by category of treatment engagement and progress**

| **In-person session number** | **Poor engagement**  **(n=63; 31%)** | **Poor progress**  **(n=80; 39%)** | **Progress**  **(n=61; 30%)** | ***P* value** |
| --- | --- | --- | --- | --- |
| 1 | 0 ± 0 | 0 ± 0 | 0 ± 0 | NA |
| 2 | 13.5 ± 10.2^a^ | 12.4 ± 8.9^a^ | 8.5 ± 3.3^b^ | 0.003 |
| 3 | 33.1 ± 31.6^a^ | 23 ± 12.3^b^ | 18.3 ± 6.1^b^ | <0.001 |
| 4 | 42.8 ± 23.3^a^ | 35.3 ± 18.9^b^ | 29.2 ± 8.4^c^ | <0.001 |
| 5 | 64.7 ± 29.3^a^ | 54.6 ± 27.2^b^ | 47.1 ± 10.5^b^ | 0.002 |
| 6 | 73.7 ± 25^ab^ | 79.4 ± 44^a^ | 65.5 ± 12.8^b^ | 0.0495 |
| 7 | 95.4 ± 14.5 | 111 ± 44.2 | 98.2 ± 18.3 | 0.06 |
| 8 | 135.4 ± 15.6^ab^ | 151 ± 49.2^a^ | 131.7 ± 25.3^b^ | 0.02 |
| 9 | 149.0 ± NA^ab^ | 189.7 ± 55.9^a^ | 164.2 ± 30.5^b^ | 0.006 |

Abbreviations: NA, not applicable (SD is NA because only 1 participant attended session 9 in the poor engagement category).

^a, b, c^ Different superscripts denote statistically significant differences between categories.
