# Supplementary material for: Variability in engagement and progress in efficacious integrated collaborative care for primary care patients with obesity and depression: Within-treatment analysis in the RAINBOW trial
Source: PLoS One. 2020 Apr 21;15(4):e0231743. doi: 10.1371/journal.pone.0231743 (PMC7173791; doi:10.1371/journal.pone.0231743)
Supplement: S8 Appendix — (DOCX) [file pone.0231743.s008.docx]

**S8 Appendix. Joint and separate cluster analysis of weight and PHQ-9 trajectories among intervention participants who had at least one self-monitored weight measure and one PHQ-9 score in all 4 quarters (n=88)**

**PHQ-9 scores**

**% weight change from baseline**

|  | **Panel A: Conjoint cluster analysis** | **Panel B: Separate cluster analysis** |
| --- | --- | --- |
|  | **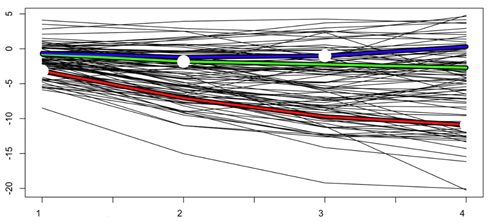**  **A**  **C**  **C**  **B** | **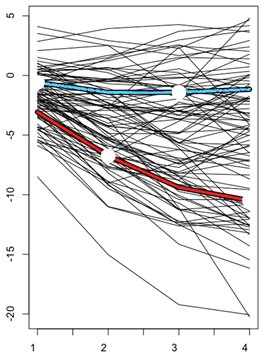**  **1**  **1**  **2**  **2** |
|  | **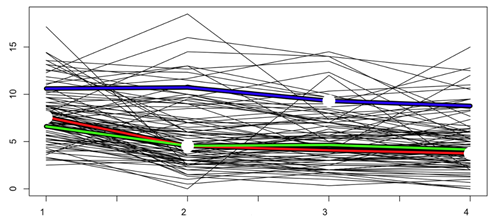**  **C**  **C**  **B**  **A** | **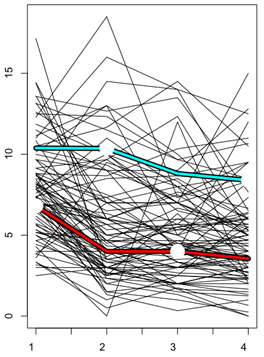**  **1**  **1**  **2**  **2** |
|  | **Quarter** | **Quarter** |
