# Supplementary material for: Variability in engagement and progress in efficacious integrated collaborative care for primary care patients with obesity and depression: Within-treatment analysis in the RAINBOW trial
Source: PLoS One. 2020 Apr 21;15(4):e0231743. doi: 10.1371/journal.pone.0231743 (PMC7173791; doi:10.1371/journal.pone.0231743)
Supplement: Attachment — Submitted filename: Response to reviewer comments_R2_FINAL.docx [file pone.0231743.s010.docx]

**We appreciate the additional comments on our revised manuscript, titled “Variability in engagement and progress in efficacious integrated collaborative care for primary care patients with obesity and depression: within-treatment analysis in the RAINBOW trial.” Below please find our response.**

**Reviewer 2’s comments:**

The authors have responded in detail to the comments and made appropriate additions to the manuscript.
My remaining reservation concerns the use of significance tests on differences in trajectory parameters between clusters to prove the distinctness of the clusters (points 1 and 7). The authors replied that their method of verification was the same as that used by Babbin et al (Ref. 31):
“We used a similar method that others have used (Babbin et al., Multivariate Behav Res. 2015, 50(1):91-108) to do cluster analysis and to verify that the derived clusters differed with regard to the regression coefficients of individual trajectories within the clusters.”
As I understand Babbin’s paper, no significance tests were performed on trajectory coefficients between clusters. Cluster-mean time-series parameters were displayed (Tab. 2), but without tests. The distinctness of the clusters was verified by displaying and testing cluster mean values of baseline variables which were not involved in the clustering (Tab. 3). This is an important difference to the method of the present study, in which the tested parameters concern the trajectories of the same variable used to create the clusters.
Therefore, I maintain that these significance tests do not prove the distinctness of the clusters. Even if one created purely random data from a single distribution, clusters could be found, and such clusters would show significantly different mean values of variables derived from those variables used to create the clusters. A proper assessment of distinctness should use a method described in the literature under the key-term ‘cluster validity’. In the absence of such an analysis, claims of distinctness and significance should be toned down.

**Authors’ response:** As suggested by the reviewer, we toned down claims of the distinctness of clusters in the methods and results (lines 177-179, 233-237, and 257-261). Additionally, we emphasized the need for future validation studies with independent samples in the discussion. As described in the methods, cluster analysis was performed using the k-means method and the optimal number of clusters was decided using a combination of criteria, including Pseudo F statistic (a relatively large value), R-squared value (a peak that flattens with additional clusters), Cubic Clustering Criterion (≥2), and cluster size (≥10 participants). Although we currently do not have an independent sample to validate the 3 clusters of weight loss and the 3 clusters of depression symptoms, we stated in the discussion section that “The 3 weight loss patterns identified in the current study were similar to those found in previous weight loss studies.[34, 35]” Validation of the clusters of weight loss and depression symptoms is needed using independent samples in future studies. We emphasized this point in the discussion of the limitations (lines 387-390): “First, because of the post hoc nature of the analyses the findings need to be replicated in future studies. Second, the weight change and PHQ-9 score clusters may be specific to the study data. Therefore, future studies of independent samples are needed to verify the external validity of the results.”
